# Leveraging Dynamic Heterogeneous Networks to Study Transnational Issue Publics. The Case of the European COVID-19 Discourse on Twitter

**DOI:** 10.3389/fsoc.2022.884640

**Published:** 2022-06-30

**Authors:** Wolf J. Schünemann, Alexander Brand, Tim König, John Ziegler

**Affiliations:** ^1^Institute of Social Sciences, Hildesheim University, Hildesheim, Germany; ^2^Institute of Computer Science, Heidelberg University, Heidelberg, Germany

**Keywords:** dynamic networks, heterogeneous information networks, COVID-19, European public sphere, discourse analysis, Twitter, transnationalization

## Abstract

The ongoing COVID-19 pandemic constitutes a critical phase for the transnationalization of public spheres. Against this backdrop, we ask how transnational COVID-19 related online discourse has been throughout the EU over the first year of the pandemic. Which events triggered higher transnational coherence or national structuration of this specific issue public on Twitter? In order to study these questions, we rely on Twitter data obtained from the TBCOV database, i.e., a dataset for multilingual, geolocated COVID-19 related Twitter communication. We selected corpora for the 27 member states of the EU plus the United Kingdom. We defined three research periods representing different phases of the pandemic, namely April (1st wave), August (interim) and December 2020 (2nd wave) resulting in a set of 51,893,966 unique tweets for comparative analysis. In order to measure the level and temporal variation of transnational discursive linkages, we conducted a spatiotemporal network analysis of so-called Heterogeneous Information Networks (HINs). HINs allow for the integration of multiple, heterogeneous network entities (hashtags, retweets, @-mentions, URLs and named entities) to better represent the complex discursive structures reflected in social media communication. Therefrom, we obtained an aggregate measure of transnational linkages on a daily base by relating these linkages back to their geolocated authors. We find that the share of transnational discursive linkages increased over the course of the pandemic, indicating effects of adaptation and learning. However, stringent political measures of crisis management at the domestic level (such as lockdown decisions) caused stronger national structuration of COVID-19 related Twitter discourse.

## 1. Introduction

Scholarly research across disciplines has shown great interest in the transnationalization of social communication through digital media. Against euphoric expectations of a “death of distance” (Cairncross, 2001) in the wake of the revolutionary period of Internet development and (again) the emergence of social networks, there is an ongoing dispute including many skeptical voices that point to a panoply of structuring factors at the national and regional scale—be it linguistic or other cultural conditions, geographical proximity or institutional factors like media markets and political systems (Straubhaar, 1991, 2010; Taneja and Webster, 2016). However, the COVID-19 pandemic has tremendously altered the basic conditions of social life across the world. It likely constitutes a critical phase for the transnationalization of public spheres. While contact and travel restrictions have strongly affected physical mobility, especially palpable in otherwise “borderless Europe” (Opiłowska, 2021), digitalization has drastically reduced the gravity with which those changes could impact routines of social communication. The availability of digital means of communication and the increased use of digital technology during the pandemic have potentially compensated for many cuts into the social fabric, especially with respect to cross-border communication. Therefore, given that the pandemic has clearly boosted digital connectivity, one could also expect it to serve as a facilitator and driver for transnational communication.

When looking at the social effects of the pandemic from the perspective of transnationalization research, there is another fundamental alternative to be studied. While, on the one hand, news and discussion about the pandemic dominate political discourse worldwide and have thus exhibited if not produced so-called “overlapping communities of fate” (Held, 1997) arguably to a greater extent than ever before, there are, on the other hand, social and political reactions to the disease that have been interpreted as relapses into national egoisms. Central measures taken in response to the pandemic have shown a pre-dominant national logic of crisis management, emphasizing the organizational needs of institutionally pre-disposed communities of place. Against this backdrop, we ask how transnational COVID-19 related online discourse has been. Do we observe new trends toward transnationally integrated social communication and discourse? Or do we see more nationally structured debates and communicative insulation driven by institutional nationalism in political crisis management? If overarching trends are inconsistent, which correlations between the course of the pandemic and crisis management on the one hand, and the transnationality of COVID-19 related online discourse on the other can be observed? How do national user communities differ in this regard?

While the theoretical discussion has been vivid over the last decades, methodology needs further development. New approaches especially need to live up to the great opportunities that Big Data and digital methods provide. With the methodological approach that we present in this paper, we make innovative use of a particularly rich resource of Twitter data (TBCOV multilingual COVID-19 Twitter dataset) and methods of digitally enhanced network analysis at scale. In particular, our approach allows for an integration of different kinds of discursive linkages (e.g., shared URLs, retweets, mentions, hashtags, named entities) into a Heterogeneous Information Network (HIN). HINs are defined as directed graphs which consist of multiple types of objects and connecting relations. These discursive linkages fall into two classes that we describe as topical and referential. To answer our research questions, we study HINs over time, allowing us to find valuable explanations for the variation observed in our data. Studying communication on social media platforms like Facebook or Twitter is a well-established research method in the social sciences (Edelmann et al., 2020; Özkula et al., 2022). This includes empirical research interested in questions of transnationality or transnationalization (Deutschmann, 2022). While being inspired by those pieces of related research, our approach turns toward a discourse-oriented methodology by concentrating on the variety of discursive linkages rather than social ties. In addition, our HIN-based approach allows for a flexible and temporally aware multi-dimensional modeling of discursive linkages, going beyond frequently applied network analytical methods.

As to the scope of our analysis, we deliberately focus our study on Europe for both pragmatic and theoretical reasons. For one, reducing the data allows us to conduct this resource-intensive research in reasonable time. Moreover, we can contribute to the ongoing debate about the European public sphere, which has been relevant for both the academic and political world for more than two decades. In the following section, we locate our study within the wider field of relevant research. In Section 3, we derive our hypotheses. Afterwards, we describe our data and methods in Sections 4, 5. Our results will be presented in Section 6 and discussed afterwards (7), before we summarize the most important findings in a short concluding section. Apart from its contribution to the ongoing debate on transnationalization within and outside of Europe, this paper advances the field of digital methods by introducing our novel, HIN-based methodology to the study of social networks and online discourse.

## 2. State of Research

### 2.1. COVID-19 and Transnationality

For the longest time of human history, infectious diseases and transnationality have found themselves in a notoriously difficult relationship. It is common knowledge in medical science and public health that physical mobility is a key driver for the spread of infectious diseases like COVID-19—a crucial insight that has motivated contact restrictions, quarantine obligations and lockdowns as effective political measures. Social network research has traditionally contributed to explanations and predictions of the spread of infectious diseases (Klovdahl et al., 1994). This has more recently been transferred to the digital sphere based on digital communication data and computational methods (for an overview, see Aiello et al., 2020). In recent empirical research based on aggregated Facebook data, scholars have found strong correlations between social ties (i.e., social connectedness via Facebook friendship) and the regional spread of COVID-19 in the US as well as Italy (Bailey et al., 2020; Kuchler et al., 2020). Furthermore, Twitter and web news data have been used to predict COVID-19 outbreaks (Jahanbin and Rahmanian, 2020; Mellado et al., 2021). Given the local origin of a new virus and higher controllability of outbreaks at a local scale, transnational mobility is seen as responsible for the growing risks that infectious diseases constitute for an increasingly globalized world. While the expectation of a negative relationship between infectious diseases and transnational mobility is thus mainly derived from medical science and public health studies, social science research has revealed additional facets of this relationship. Most fundamentally, researchers have put emphasis on the social construction of the risk of contagion (Bury, 1986; Conrad and Barker, 2010). Previous sociological work has shown how risk perceptions related to infectious diseases tend to be discursively coupled with social attitudes or convictions like colonial attitudes, xenophobic fears or racist convictions (Bhopal, 2014). This can lead to discrimination and stigmatization of outgroups with detrimental effects on transnational mobility in general and migration in particular (King, 2002; Bhambra, 2014). Empirical studies have found evidence for such discursive tendencies with respect to earlier diseases such as Aids/HIV, Ebola or Tuberculosis (Bancroft, 2001; Monson, 2017; von Unger et al., 2018, 2019), each accompanied by stereotypical fears toward (foreign) minorities detectable in different kinds of media discourses, including social media like Twitter and Facebook (Roy et al., 2020). Similar findings have been made recently with respect to COVID-19 and anti-Asian sentiment (Li and Nicholson Jr, 2021; Reny and Barreto, 2022). Judged from such perspectives alone, the expected effects of a global pandemic on transnationality can only be negative. Yet, the development of communication technologies in the age of digitalization has freed social communication (and connectedness) from its ontological relationship with physical mobility to a degree that it can now be regarded as an independent dimension of "transnational human activity" with different rules and expectations (Deutschmann, 2022). Therefore, the question about the effects of a global pandemic on transnational communication and discourse must be posed in a different way as it opens up new and relevant avenues for empirical research. To our knowledge, there has been no study taking up this demand by systematically studying the transnational quality of COVID-19 related online discourse so far.

### 2.2. The Ongoing Quest for the European Public Sphere

The emergence of a transnational public sphere has most prominently—and frequently—been studied with respect to the so-called European Public Sphere (Risse, 2010, 2015). The historical development and current state of Europe's political integration have driven normative and empirical expectations toward transnationalization. Empirical studies in the field have started out from different theoretical conceptions and adapted various methodologies (see Pfetsch and Heft, 2015). Besides more discourse-oriented studies (Koopmans and Zimmermann, 2010; Kantner, 2015), scholars have applied network analysis especially when studying communicative linkages in Internet communication and social networks (Koopmans and Zimmermann, 2010; Deutschmann et al., 2018; Ruiz-Soler, 2018; Schünemann, 2020; Stier et al., 2021; Wallaschek et al., 2022). While empirical scholars judged differently with respect to the fundamental question of whether there is such a thing as a European public sphere, there is some convergence around a common baseline observation. According to this insight, a European public sphere is not expected to appear “above and beyond the various national or issue-specific public spheres,” but rather through the “Europeanization of national and other public spheres” (Risse, 2015, p.17). This is relevant also for our approach, as it lends additional justification to a less demanding operationalization of transnationality by measuring discursive linkages instead of actual social ties.

### 2.3. Transnational Communication and Digital Data

The Internet and social media are transnational by design. Coming from the perspective of the “networked public sphere,” prominent scholars have predicted an extension of social communication across borders early on Benkler (2006) and Castells (2008). Internet technology and especially social media platforms would open up “electronic elsewheres” (Berry, 2010) as new places for social interaction (Papacharissi, 2015). Moreover, the structural transformations induced by digitalization would affect the concept of the public sphere as such, with a network of issue publics emerging instead of the single public constituted by traditional mass media (Bruns, 2008, p.69). For this process, Twitter plays a particularly important role as a central platform for the emergence of (*ad-hoc*) issue publics—at least in the Western Internet ecosystem (Bruns and Burgess, 2011). Methodologically, there is a broad range of measurements for transnationalization (Pfetsch and Heft, 2015). Traditionally, transnational communication flows have been assessed by network analysis (Koopmans and Zimmermann, 2010; Deutschmann, 2022) or discourse oriented studies (Koopmans and Statham, 2010; Kantner, 2015). More recently, scholars have turned toward digital trace data and computational methods (State et al., 2015; Taneja and Webster, 2016; Schünemann, 2020). However, most studies have looked at only one kind of linkage such as direct interactions, link-sharing or discourse in an isolated way and thus have not allowed for a combined perspective on different indicators of (trans-)national structuration.

### 2.4. Twitter Data and Empirical Research

Twitter is a unique data source for digital communication. Compared to other social media, data access for researchers is relatively easy and comprehensive (Özkula et al., 2022). However, there are important limitations that have to be kept in mind when using social media, and especially Twitter, data for social science research. These have been widely documented in the relevant research literature (Boyd and Crawford, 2012; Ruths and Pfeffer, 2014). The lack of representativeness in terms of the demographic characteristics of Twitter users has been discussed most broadly. Previous research has shown that social media users in general and Twitter users in particular tend to be younger, better educated and politically more liberal (Malik et al., 2015; Mellon and Prosser, 2017). While such bias is indeed likely to influence transnational communication and discourse, we would argue that this lack of representation affects our comparative study less than works that make inferences to the wider population. After all, it is precisely this subset of the population that is more likely to communicate transnationally across all countries.

Of greater relevance to our study are Twitter's geographical, cultural and linguistic biases. While Twitter is used by a large community of users across the globe, there are cultural, regional and national differences that should be taken into account. Most obviously, activity on Twitter is very unequally distributed across the world, with dominant use in the United States followed by other OECD countries (Barnett and Park, 2014). With respect to our regional focus, for example, the Reuters Institute reported that in 2020, 29 percent of the British population were Twitter users, compared to 33% in Spain, 18 % in Italy, and only 13 % in Germany (Reuters Institute, 2020). These numbers also show the strong bias toward English-speaking, and especially Anglo-Saxon, countries. Furthermore, the user base in different countries uses the platform for different purposes. Table 1 reports platform use across countries in our sample for both general purpose and news consumption. The latter are markedly lower, ranging at about half of the values for general usage. Moreover, different ratios (news in relation to any purpose) might be telling with respect to divergent usage patterns. For instance, the respective ratio is only at 0.31 for Hungary against 0.57 and 0.58 for Spain and Ireland, respectively. Cultural and national differences in how social media are used have been studied since their inception (Chu and Choi, 2010; Poblete et al., 2011; Sheldon et al., 2017; Hong and Na, 2018). International variation in social media usage patterns has been explained by cultural differences, e.g., between more individualist and more collectivist cultures (Chu and Choi, 2010; Shneor and Efrat, 2014; Sheldon et al., 2017). With respect to the method chosen for this paper, entity-based indicators for differences in usage are of particular interest here. So, for instance, frequent appearances of @-mentions and especially retweets have been interpreted as indications of a higher tendency to use Twitter for formal news dissemination. In contrast, a lesser degree of retweeting in a country sample would rather be read as showing a higher use of Twitter for conversational purposes (Poblete et al., 2011). Since country-specific general usage patterns likely affect the transnationality of COVID-19 related Twitter discourse, the respective statistics need to be taken into account (see Sections 6, 7 for results and discussion and Supplementary Figure 3 for full statistics).

**Table 1:** KOF Globalization Index 2019 (KOFGI) social dimensions, Reuters social media usage 2020 (any purpose / general usage); and Reuters social media usage 2020 (news) by country.

**Country**	**KOFGI (Score)**	**Reuters general (%)**	**Reuters news (%)**	**Country**	**KOFGI (Score)**	**Reuters general (%)**	**Reuters news (%)**
Austria	525	10	5	Italy	477	18	9
Belgium	514	13	5	Latvia	493	–	–
Bulgaria	461	16	8	Lithuania	515	–	–
Croatia	500	12	4	Luxemburg	546	–	–
Cyprus	499	–	–	Malta	511	–	–
Czechia	496	8	4	Netherlands	516	16	7
Denmark	521	12	5	Poland	458	21	11
Estonia	499	–	–	Portugal	492	15	8
Finland	514	19	8	Romania	458	16	6
France	513	16	9	Slovakia	488	7	3
Germany	524	13	6	Slovenia	481	–	–
Greece	501	25	13	Spain	497	35	20
Hungary	473	13	4	Sweden	525	17	8
Ireland	523	24	14	United Kingdom	532	29	14

Returning to a macro-level of comparison, Twitter adoption itself is likely being influenced by the extent to which a national population is culturally globalized. The KOF Globalization Index (KOFGI) shall serve as a yardstick for assessing the extent to which a country is globalized in the following sections (Gygli et al., 2019). In order to provide an aggregate measure for the social dimensions of globalization, Table 1 gives the respectively summed country scores of KOFGI for 2019. We can see at first glance that they do not significantly correlate with Twitter usage which is explainable by the fact that Twitter is only one social network among others and not the most central one for most populations.

Finally, there is a strong linguistic bias toward the English language in every global Twitter dataset. This, however, is not Twitter-specific, but rather reflects the special function of the English language for global communication—a kind of global language, especially online. As previous research has shown, English is the dominant language in cross-nationally linked online issue publics. For example, linguistic communities are more likely to be linked via English websites than direct ties, and content that is provided in other languages than English is unlikely to be recognized by international audiences at all (Hale, 2012). The effects of these phenomena on the results of our study on transnational COVID-19-related online discourse will be discussed in Section 7.

### 2.5. Heterogeneous Information Networks

So far, social science network research has not fully embraced the idea of heterogeneity. Networks in social science research traditionally grasp direct social ties between two or more actors in a form of a sociogram or various kinds of actor-entity relations in an affiliation network. This actor-centered orientation of network analysis has particularly strong foundations in the tradition and theory of social action. Yet, other academic disciplines have increasingly shown that network analysis can be mobilized to study a broad variety of relational structures, including language and knowledge (Sowa, 2014). Previous research in the field of Computational Social Sciences also highlights the need to combine computational methods and social science theories when studying social media related questions (Fernandez et al., 2018). Nevertheless, prominent studies from the field of Heterogeneous Networks do not take theories from social sciences into account. This includes relevance measures based on meta paths that are used for searching similar nodes in HINs (Sun et al., 2011; Shi et al., 2012, 2014), but also methods to cluster or classify nodes into categories (Kong et al., 2012; Sun et al., 2013). While such approaches might be feasible in a context where only non-social relationships are considered, they might not be suitable for the study of social networks. Simply resorting to “information networks” as non-social does not resolve this shortcoming. Social media data, after all, clearly involves social interactions and might as well be modeled as HIN (Sun and Han, 2013). This is confirmed by recent work that leverages the Twitter network as HIN for recommendation and classification tasks (El-Kishky et al., 2022). With our approach, we build on theoretical conceptions from social sciences and communication studies. In contrast to other research in the field, we study discursive linkages instead of direct social ties. Unlike the related field of semantic network research developed in the fields of linguistics and Artificial Intelligence (Sowa, 2014), our approach does not concentrate on the level of concepts and linguistic structures, but integrates various relevant entities, including users and messages. Finally, whereas other network analytical research has remained static, we systematically include the temporal dimension. This latter feature is of crucial relevance given the procedural character of transnationalization and the dynamic character of the pandemic.

## 3. Hypotheses

With these considerations in mind, we expect transnational discursive linkages via Twitter to intensify with the severity of the pandemic. More precisely, when comparing the major phases of the pandemic throughout its first year, we expect shares of transnational linkages to go up during the so-called waves of the pandemic. Thus, for the macro-level perspective, we formulate our first hypothesis as follows:

H1: We expect the share of transnational discursive linkages on Twitter to positively correlate with the severity of the pandemic.

Despite the inarguably transnational potential of social media communication, scholarly research has questioned the more substantial effects with respect to patterns of social connectedness and mass media publics that both still seem to be predominantly structured along national lines (Straubhaar, 2015; Bailey et al., 2020). While more cosmopolitically oriented elite actors—which are evidently overrepresented in most Twitter samples—practice transnational communication, the majority of users is still oriented toward the mainstream media and their national media logic. Moreover, not only are general social connectedness and media publics inherently structured along national lines, but the patterns observable in crisis management even across Europe have been critically discussed as exhibiting regrettable forms of neo-nationalism (Wang, 2021). Related to this discussion, it is important to keep in mind that especially political decisions on stringent measures have affected societies across the world at different times and to a different extent over the course of the pandemic. Such events are thus likely to produce peaks in society-specific communication, potentially inducing greater national structuration of public discourse and thus declines in transnational communication. Therefore, measured on the basis of daily events and its immediate effects, we expect the shares of transnational discursive linkages to decrease with the implementation of crisis management measures, such as lockdowns, in a certain country. Therefore, we formulate our second hypothesis as follows:

H2: We expect transnational discursive linkages to negatively correlate with restrictive national measures.

Returning to the macro-perspective, overall effects of the pandemic on transnational discourse are not necessarily stable over time. Rather, we can expect processes of adaptation and learning over the course of the pandemic. Therefore, we can expect to observe variation between the two COVID-19 waves in our research period. Especially the first wave of the pandemic accompanied by the first national lockdowns might have produced some kind of shock-induced paralysis with people suddenly restricted to their homes, many social relations temporarily cut, and experiences of a particular state of exception. Therefore, we expect more national communicative activity during the first wave. In contrast, during the second wave, after having adapted to the Corona situation, including restrictive measures, and having established new digital ways to connect also in spheres where this has not been common beforehand, discursive linkages might have become more transnational over the development of the pandemic. Moreover, as our measurements are influenced by Twitter routines, one can assume that COVID-19 related communicative routines and codes have been established over time, facilitating discursive cohesion with the pandemic evolving. Taking these reflections into account, we formulate the following hypothesis:

H3: We expect the share of transnational discursive linkages to positively correlate with the duration of the pandemic situation due to processes of learning and adaptation.

Finally, with respect to international variation, we expect countries that are less globalized with respect to socio-cultural indicators to show less alignment with global COVID-19 related discourse and thus have lower shares of transnational discursive linkages over all periods studied. Therefore, we formulate our final hypothesis as follows:

H4: We expect the share of transnational discursive linkages to positively correlate with the extent to which a country is globalized in socio-cultural terms.

## 4. Data

For our analysis, we chose the TBCOV Twitter dataset, a corpus of over 2 billion multilingual tweets posted between February 1st, 2020 and March 31st, 2021 (Imran et al., 2021). For now, TBCOV constitutes the most comprehensive dataset of worldwide Twitter communication on the pandemic. The TBCOV team collected tweets based on more than 800 multilingual query terms. There are crucial advantages of this dataset compared to similar resources (Dimitrov et al., 2020), namely that TBCOV is a multilingual dataset which is not restricted to English-language tweets. This makes it more balanced and less biased toward Anglo-Saxon communication flows. Moreover, tweets are geolocated with a multi-tier geolocation approach, using geotagged information, a lookup for user location entries and for elements with location information extracted from the body of the message via the Nominatim API. After processing, their dataset consisted of messages from 87 million unique users, across 218 countries, writing in 67 languages (Imran et al., 2021). We rehydrated the data for our subset of tweets located in one of the EU-27 countries or the United Kingdom. We further reduced the dataset by a selection of research periods representing the different phases of interest, namely April (1st wave), August (interim), and December (2nd wave). We understand the interim period as a relative reference period that helps us assess the effects of the pandemic waves on the transnationality of Twitter discourse. Our remaining dataset after rehydration through the Twitter APIv2 consisted of 51,893,966 tweets. Relevant discursive linkages, such as URLs, Hashtags and User Mentions, were extracted from the Twitter API, or, in the case of named entities, provided by the TBCOV dataset. Deviations in data from the full TBCOV dataset were mostly assignable to Twitter-initialized factors like bans.^1^

In order to determine the level of restrictions in the respective countries during our research period, we use the Covid Stringency Index. This index is a publicly available, day-wise composite measure based on indicators like school closings, work related restrictions and travel bans, scaled between 0 (no relevant restrictions) to 100 (strictest measures) offered by the Oxford COVID-19 Government Response Tracker project (OxCGRT) (Hale et al., 2021). It consists of a weighted summation of nine ordinally scaled indicators, whose numbers increase from recommendation to obligation of restrictions. The OxCGRT coded these indicators individually according to publicly available sources, e.g., news articles, press releases, and briefings.^2^ A more detailed description of the indicator's development for EU-27 plus Great Britain is offered in the Supplementary Figure 2.

We obtained data on Twitter usage per country from the Digital News Report 2020 issued by the Reuters Institute for the Study of Journalism (Reuters Institute, 2020). Its yearly report is based on an international representative survey covering 21 of the 28 countries in our study (see Table 1). The dataset provides percentage values for Twitter usage for a) any purpose and b) news per country.

Finally, for assessing socio-cultural globalization per country, we rely on a combination of “social dimension” indicators taken from the KOF Globalization Index in its revised version for 2019 (Gygli et al., 2019). We obtained data for the six composed sub-indicators Interpersonal Globalization (de facto and de jure), Informational Globalization (de facto and de jure), Cultural Globalization (de facto and de jure), (each ranging from 0 to 100) and calculated a summarized score (see Table 1).

## 5. Methods

### 5.1. Measuring Transnationality Through HIN-Based Methodology

Most network analytical studies on transnationality or transnationalization have analyzed actual activity. Thereby, researchers using digital communication data normally set a much higher threshold for relevant transnationality due to its exclusive orientation toward user interactions. We would argue that reading Twitter's default options for the creation of communicative linkages as ready-made relations upon which to construct a sociogram overestimates the real-world meaning of such platform-induced interactions. Furthermore, it disregards other, more subtle but still relevant, connective patterns in online discourse. Finally, the actual use of a platform's built-in connective features depends more strongly on pre-existent social network constellations and their reflections in platform membership or user hierarchies than the more balanced set of discursive linkages that we include in our HINs. In effect, this makes our measurement less prone to underestimate transnational alignments. Moreover, our approach is less bound to and biased by Twitter's affordance architecture.

By reorienting network analysis toward discourse research, our HIN-based approach puts emphasis on shared knowledge structures and discursive patterns instead of mediated user interactions. However, instead of representing webs of knowledge based only on one class of co-occurring linguistic or other signifiers (e.g., words), our approach allows to also include actors, documents (in our case tweets) and various kinds of automatically extractable entities as nodes in the overall network. While this fundamental feature makes our approach applicable to a wide range of research questions in the study of social communication beyond Twitter, transnationalization or the issue at hand, it is of course important to specify the linkages included in a HIN from case to case depending on the research questions. In the following sub-sections we present some basic reflections on the dimensionality of our linkages and make some conceptual clarifications before we outline our methodological approach in greater detail.

### 5.2. Discursive Linkage Dimensions

Similar to other network analyses, our HIN-based methodology bears the risk of flat ontologies with respect to the actual quality of the relations observed in the network. To determine what kind of connection a certain meta path constitutes in the real world is not always a trivial task. Relevant entities for our study certainly differ with respect to the kind of relationship they establish. Using the same named entity in a tweet as another user does somewhere at the other end of the world (or in the next village) constitutes a low-threshold linkage in comparison to sharing the same URL, not to mention actual user interactions. However, since such differences in frequency and likelihood cannot be easily translated into a differentiated metric, we have not weighted the entity-specific instances of our linkage types for our aggregate measure. In order to provide more differentiated information, we nevertheless present disaggregated measures and consider the individual types of linkages in our dataset. At a conceptual level, we distinguish between referential linkages via retweets or URLs and topical linkages via named entities, hashtags or @-mentions.

#### 5.2.1. Referential Linkages

Referential linkages stand for a connection between two users due to the reference they make to the same content. While retweeting is the most frequently used in-built function for making reference to other content within the Twitter platform, sharing URLs constitute the standard practice of hypertext referencing within the much wider web-based media environment.

*Retweets*: In most Twitter-based communication studies using network analytical methods, retweets are conceived as direct links between the retweeting user and the user that has been retweeted (Ruiz-Soler, 2018; Stier et al., 2021). While such standard operationalization of social ties in Twitter research seems straight forward, it is important to keep in mind that Twitter ties, including retweets, are relatively weak (Takhteyev et al., 2012). Moreover, retweeting is heavily conditioned by both Twitter's affordance architecture and the pre-existent social relations partly represented in follower networks. Conceiving retweets as sharing of third party content as we do in this study has the advantage to not overestimate social ties that retweet interactions otherwise might suggest. In addition, retweets as entities can be better aligned to the overall taxonomy of linkages. We detected retweets based on the metadata available in the TBCOV dataset.

*URLs*: Hyperlinks represented by URLs as automatically extractable entities point to the relational core feature of web technology that platforms such as Twitter also rely on (Benkler, 2006). The analysis of hyperlinking patterns is standard practice in the study of online communication and has played a major role in earlier periods of internet development (Adamic and Glance, 2005; Hale, 2012). It is still a relevant approach and transferable to social media platform communication (Jacobson et al., 2016; Schünemann, 2020). Co-sharing of URLs establishes a connection between two users when referring to the same content in the wider universe of the web. Therefore, it allows to integrate referential linkages that are not Twitter-specific or dependent on the platform. Accordingly, hyperlinking has been taken as a proxy to measure awareness of media content across national or linguistic borders in previous research (Barnett et al., 2011; Taneja and Webster, 2016). We obtained the expanded URLs when rehydrating tweets via the Twitter APIv2.

#### 5.2.2. Topical Linkages

Topical linkages group a second dimension of discursive connections. They indicate that two users in a pair of tweets deal with the same issue or topic. Entities assigned to this dimension are named entities, hashtags and @-mentions.

*Named entities*: At the conceptual level, named entities are the symbolic representations of various kinds of real-world objects or entities such as persons, locations or organizations, that can be automatically extracted from tweet text. Co-usage of named entities is a low-threshold discursive linkage establishing a connection between two users that in a pair of tweets speak about roughly the same things. Named Entity Recognition (NER) is a standard procedure in information extraction. We obtained named entities as metadata directly from the TBCOV dataset. NER is particularly error-prone and must be scrutinized accordingly. However, given the sheer amount of named entities extracted, we are optimistic that error rates are negligible with respect to our overall indicator. Nevertheless, it is important to keep the low threshold for matches in mind when interpreting absolute numbers of linkages of this type. As this holds true for this type of linkages regardless of the national/transnational quality, we still trust our measurement based on the relative weight of transnational linkages.

*Hashtags*: The use of hashtags is a very prominent built-in function of the Twitter platform by which users themselves can ascribe their tweet message to a broader topically oriented debate. While NER-based linkages can be regarded as the least deliberate discursive events that we include in our taxonomy, hashtags represent the opposite of this spectrum, given that users consciously relate their messages to ongoing debates. Thus, hashtag usage is completely interwoven with the platform's affordance architecture and the sociotechnical environment it constitutes. However, given their increased visibility in general public spheres and its platform-induced value for strategic communication, hashtags are of great relevance for online discourse analysis. Studying hashtag occurrence and co-occurrence has become a standard approach in related research and hashtags themselves are taken as markers of online issue publics (Steinskog et al., 2017; Eriksson Krutrök and Lindgren, 2018; Haunschild et al., 2019). In our taxonomy, co-usage of hashtags constitutes a platform-specific discursive linkage between two users using the same hashtag in a pair of tweets. Hashtags were obtained when rehydrating tweets via the Twitter APIv2. As certain hashtags had served as query terms for TBCOV's initial data collection, we have disregarded all linkages produced via these hashtags.

@*-mentions*: Almost everything that has been said about retweets could also be repeated for @-mentions. @-mentioning is an in-built functionality of the platform, it is thus frequently used in Twitter communication and highly conditioned by Twitter's affordance architecture. As such, it is arguably less dependent on (though certainly influenced by) pre-existent follower networks than retweets, as users do not need to come across third party content but can simply type the user handle or the name of another user and wait for suggestions made by the algorithm to select the right handle. On the other hand, in a sociogram based on Twitter data, @-mentions would constitute an even weaker tie than retweets as they can have various meanings. In computational social science, however, @-mentions have been used as an indicator for direct communicative linkages (Stier et al., 2021). Against this backdrop, it might seem counter-intuitive that we subsume @-mention based linkages under topical instead of referential linkages. We argue, however, that as we do not include any direct communicative linkages between two users in our basic heuristics, co-mentioning of a third party can serve as an indicator for talking about the same things (i.e., persons or events). In this respect, @-mentions are arguably closer to named entities (type ‘person') than to retweets. @-mentions were obtained when rehydrating tweets via the Twitter APIv2.

### 5.3. Model Descriptions

**Figure 1:**
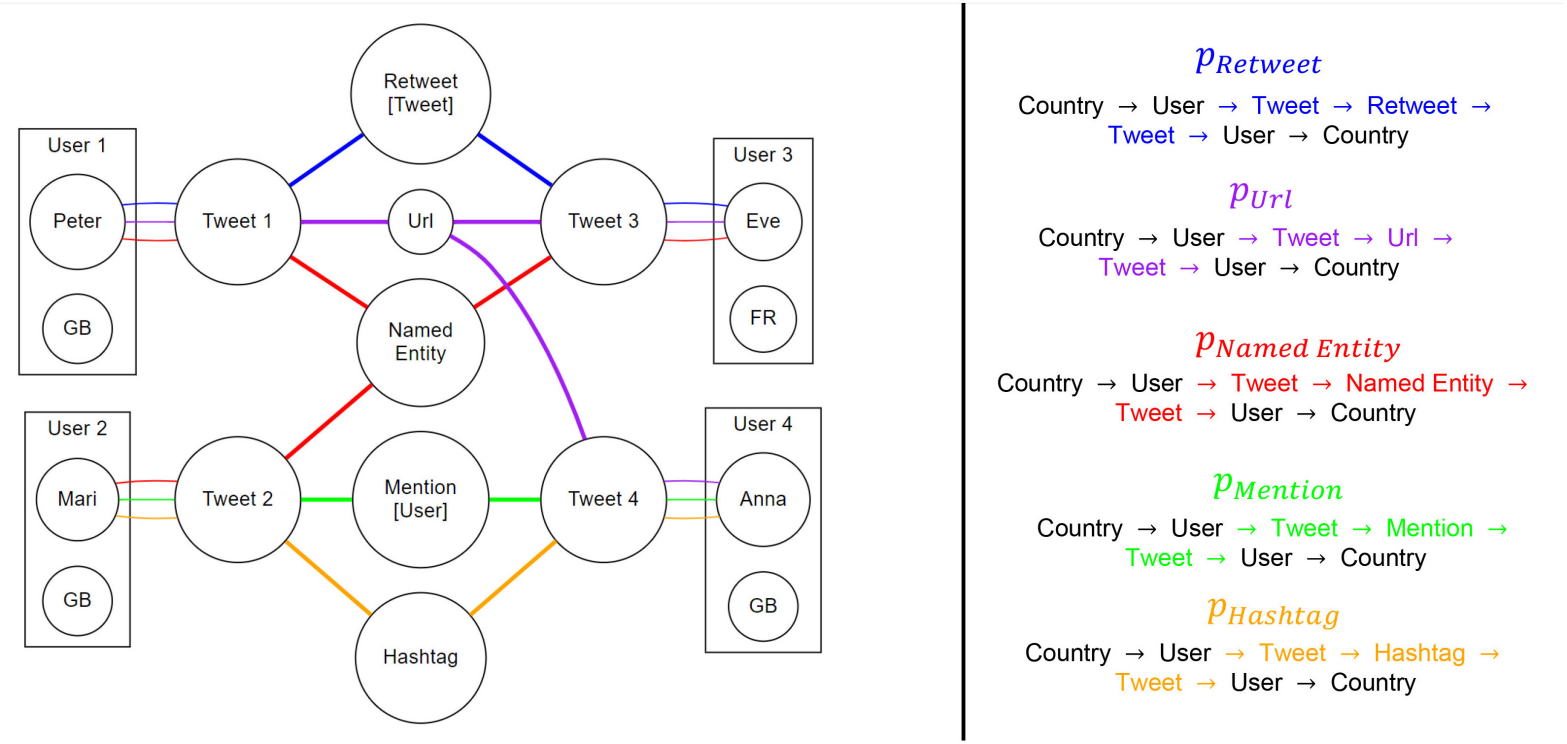
Structure of considered connections and corresponding meta paths for each type. National meta paths are constituted via connections between users from the same country, e.g., for *p*_*Hashtag*_ from Mari to Anna (both located in Great Britain) and for *p*_*Named Entity*_ from Peter to Mari. Transnational paths are e.g., *p*_*Url*_ from Peter to Eve (from Great Britain to France).

We apply Heterogeneous Information Networks (HINs) to COVID-19-related communication on Twitter. Following Sun et al. (2011) we understand a HIN as a directed graph which consists of multiple node and/or edge types, representing multiple types of objects or multiple types of relations between objects. In formal notation, we can describe a HIN as a graph *G* = (*V, E*), consisting of nodes/vertices *V* and edges/links *E*, with an additional node type mapping of ϕ: *V* → *A* (node types) and a link mapping of ψ: *E* → *R* (edge types). Further, meta paths *P* which describe paths on the graph have the form of $A_{1}\overset{R_{1}}{\to }A_{2}\overset{R_{2}}{\to }\ldots \overset{R_{l}}{\to }A_{l+1}$ (Sun and Han, 2013) following the given network scheme *T*_*G*_ = (*A, R*). Using a hashtag, retweeting another message or sharing a URL are all regarded as discursive events and users are related via these discursive events they co-produce. Thereby, we establish discursive linkages between users. For instance, a user whose message has been retweeted by another user does not constitute a node in our network as such. Instead, we take the retweet information as central connector of a multi-hop linkage type whose instances connect a user to all other users retweeting the same message. This allows us to align all entity-specific linkage patterns to one taxonomy of similarly constructed multi-hop linkage types (meta paths) in our HIN (see Figure 1).

Among all possible meta paths *P* in our network, we consider only those that correspond to referential or topical linkages outlined above and refer to them as *P*′. Those meta paths follow the structure visualized in Figure 1 and can be differentiated by their connecting central node which is part of the set {*Retweet, Hashtag, Mention, Named Entitiy, Url*}. Accordingly, we denote the different sets of meta paths as $P_{Retweet},P_{Url},P_{NamedEntity},P_{Hashtag},P_{Mention}\subseteq P^{\prime }$ and name the starting and end nodes as *v*_*s*_ and *v*_*e*_. Given *C* as set of countries present in the dataset and *V*_*c*_ as set of country nodes, we can further state that *v*_*s*_, *v*_*e*_ ∈ *V*_*c*_ for all of our analyzed meta paths. We get the actual country of a node via a mapping σ: *V*_*c*_ → *C*. Therefore, to determine whether a given meta path *p* represents a national interaction (as opposed to a transnational), we define π(*p*) = δ(σ(*v*_*s*_), σ(*v*_*e*_)) with δ being the Kronecker-Delta and *p* ∈ *P*′ as such indicator. Now, to calculate the transnationality score τ for a set of meta paths *P*′ that start and end at country nodes, we leverage π as follows: $\tau \left(P^{\prime }\right)=1-\frac{\underset{p\in P^{\prime }}{\sum }\pi \left(p\right)}{\left|P^{\prime }\right|}$. Intuitively, this represents the fraction of meta paths that represent interactions between users of different countries. From the given definition one can conclude that 0 ≤ τ ≤ 1 holds true in all cases. Furthermore, for the temporal dimension of the discursive interaction described by a meta path, we resort to the publishing dates of the tweets in such a path. Meta paths are therefore taken into account within all time windows in which those dates fall. To test our hypothesis about a causal relationship between the stringency of crisis management measures and transnationalization, we rely on multilevel regression modeling. Here, we estimate six different models (one for each meta path type and a composite) with the stringency index as our central independent variable. Additionally, we include a month-wise term. This allows to control for general seasonal effects which may play a role regarding the transnational communicative patterns (e.g., summer vacations, Christmas). Additionally, we specify a country-wise random effect variable to control for unobserved heterogeneity and different approaches to restrictions. Finally, we control for meta path-specific effects in the composite model number six. To explore the effect of the level of national restrictions on τ, we estimated linear mixed effects models for each meta path of the structure: $\tau_{ip}\sim N\left(\mu_{p},\sigma_{p}^{2}\right)$, which defines the assumption of a normal distributed dependent variable which can be modeled by estimating μ = α_*c*[*ip*]_ + β_1*p*_(stringency index) + β_2*p*_(month) for the linear combination of terms and additionally normal distributed separate intercepts $\alpha_{cp}\sim N\left(\mu_{\alpha_{cp}},\sigma_{\alpha_{cp}}^{2}\right)$ for each country *c* and each meta path *p*. For the full model we build on a similar structure appending our general formula to explain $\tau_{i}\sim N\left(\mu ,\sigma^{2}\right)$ with a meta path-specific term to μ = α_*c*[*i*]_ + β_1_(stringency index) + β_2_(month) + β_3_(metapath), dropping the separation according to the meta path type in the random effects parameter $\alpha_{c}\sim N\left(\mu_{\alpha_{c}},\sigma_{\alpha_{c}}^{2}\right)$ for each country *c*. Finally, in order to assess the impact of cultural globalization on transnational linkages in COVID-19 related Twitter discourse, we calculate Spearman's Rank correlations between the social dimensions of KOFGI and the aggregated indicator value for each country, summarized for the whole time period. We rely on a non-parametric correlation method due to non-normal distributions in the used variables.

## 6. Results

### 6.1. Comparative Results: Time Periods

**Figure 2:**
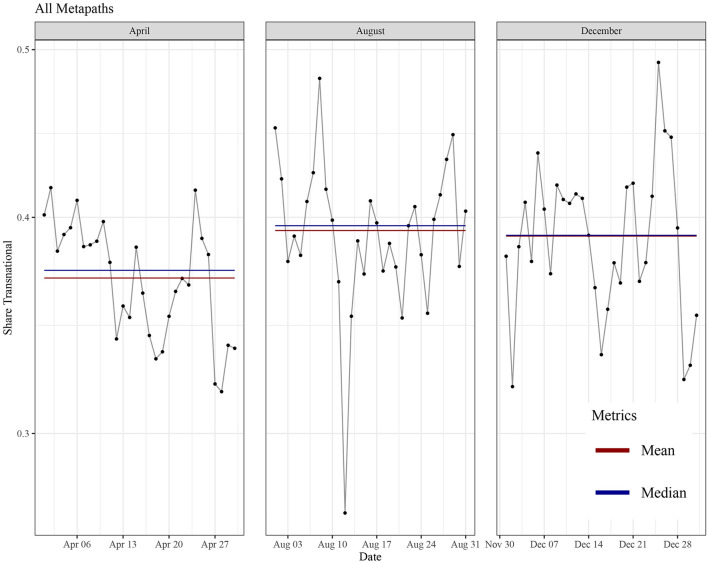
Share of transnational linkages aggregated over all meta paths. The subfigures show the development of the share on a daily base (black line), monthly mean (red line) and monthly median (blue line). for the 3 months in the sample. The shares are presented on a logarithmic scale (y- axis) to better account for different magnitudes.

**Figure 3:**
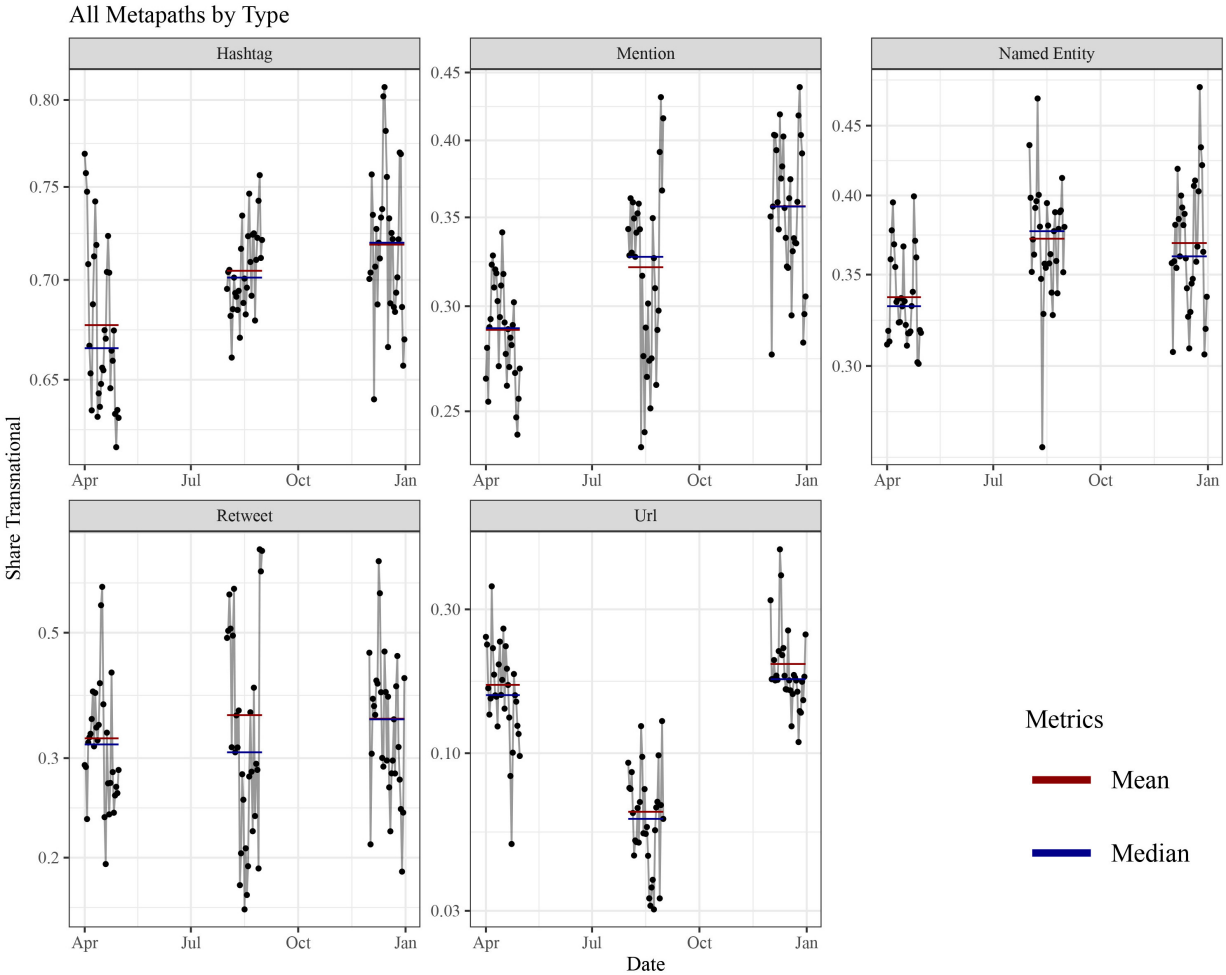
Share of transnational linkages for all meta paths. The subfigures show the development of the share on a daily base (black line), monthly mean (red line) and monthly median (blue line), differentiated by type of path. The shares are presented on a logarithmic scale (y- axis) to better account for different magnitudes.

First, we consider the aggregate indicator for transnational discursive linkages (all meta paths) per time period (1st wave, interim, 2nd wave). In total, we found 5,216,112,060,389 instances of meta paths that constitute the basis for the calculation of indicator values (1st wave: 2,213,876,499,502; interim: 1,053,010,119,563; 2nd wave: 1,949,225,441,324). A meta path-wise summary is depicted in Supplementary Table 1. Figure 2 presents the share of transnational linkages per day for each of the phases in a line plot. Values are relatively fluid, mostly ranging from about 32–45% of all linkages (95% of all observations). A total minimum beyond this range is reached on a single day in August (interim period) with only about 27% total share of transnational linkages, a maximum at about 49% on a single day during the second wave. While there are no clear trends observable within one of the phases, there is a general upward movement with aggregate measures (mean and median) ranging higher for the interim than for the 1st wave and a more or less stable level of aggregate measures between interim and 2nd wave. Both the upward development toward the interim period and stagnation toward the second wave question hypothesis 1. The upward trend over the course of the year, however, lends support to hypothesis 3, as it corresponds to the expectation of adaptation and learning of communicative routines and the establishment of common discursive patterns during the pandemic which had not been effective in the first wave. The global share of transnational linkages is aggregated over all meta paths. The general impression can be differentiated by disaggregating the global indicator and by looking at the respective timelines for each meta path-specific subindicator. This is portrayed in Figure 3. The value ranges differ significantly between the subindicators. Hashtags produce the highest shares of transnational linkages, which was expected given the essential role of hashtags for the transnationally integrated affordance architecture Twitter provides as a global platform. The other topical linkages seem closer aligned to nationally structured discourses as transnational linkage shares are generally lower. As to the referential dimension, we see similar levels of values with wider ranges for retweets. Finally, transnational linkages realized via URLs are significantly lower, which indicates stronger dependence on nationally structured media logics and public spheres. Moreover, comparing the ranges of values over the different phases of the pandemic, an upward movement of transnational linkage shares can be identified as the clearest developmental pattern. It can be observed for all entities directly related to Twitter (hashtags, @-mentions and retweets) as well as named entities. For both retweets and named entities, however, the development appears to be mitigated with more or less stagnation of aggregate measures between the interim period and the 2nd wave. This might be explained by their closer alignment to either nationally structured discourse (named entities) or pre-existent Twitter follower networks, making changes in retweeting practice arguably more inert than in other kinds of linkages. Disaggregation also helps to understand that a steady upward development for the overall indicator seems to be impeded by named entities, which due to the masses of linkages produced via this entity have a huge weight in the overall indicator. The upward trend, observed as the clearest pattern in disaggregated results, lends additional support to our hypothesis 3. In this vein, adaptation and learning can indeed be observed over the course of the pandemic, with new communicative routines and discursive patterns successively established when dealing with the global state of exception—especially on a platform like Twitter. This overall pattern is somewhat contrasted by the timeline of URL-based linkages. While it shows an upward development between the two waves, values are significantly lower during the interim period, producing a U-curve with the valley during the mid-term period. Interestingly, the subfigure would meet our intuitive expectation formulated in hypothesis 1, as it reveals that with the severity of the pandemic public attention rises for international sources and web content shared via social media, whereas this transnational issue attention is likely to be reduced during phases of relative calm.

### 6.2. Stringent Measures and Transnationality-Multilevel Model (Mixed Effects Models)

For our assessment of the effects of crisis management measures taken by political authorities, we exerted a multilevel analysis that models the relationship of stringent measures at the national level with the transnationality of Twitter discourse. Table 2 shows the regression estimates with respect to the stringency of crisis management measures (compare Supplementary Figures 1, 2) as independent variable and the meta path-specific indicators for transnationality as well as the aggregate indicator (all meta paths) as dependent variables. The fitted linear mixed effects model to explain the influence of stringency (10 point steps) on the share of transnational communication via hashtag paths (model 1) shows a statistically significant and negative effect (β = –1.11e-03, *p* = 0.006) of tightening restrictions. Furthermore, we see significant effects on a month-wise level with a negative effect (β = –3.12e-03, *p* = 0.041) for the interim period and for the 2nd wave (β = –1.33e-03, *p* = 0.141). For mentions (model 2), we observe a statistically significant and negative effect (β = –0.01, *p* < 0.001) of stringency and similar observations for hashtags with negative effects for the interim (β = –0.05, *p* < 0.001) and the 2nd wave (β = –0.03, *p* < 0.001). Regarding named entities (model 3), we observe the same direction for stringency (β = –1.62e-03, *p* < 0.001) and for the interim (β = –9.96e-03, *p* < 0.001), but no significant difference for the 2nd wave compared to the level of transnational linkages during the 1st wave (β = –1.08e-03, *p* = 0.085) ceteris paribus. For retweets (model 4), we continue to see statistically significant and negative effects for stringency (β = –0.01, *p* < 0.001), interim (β = –0.07, *p* < 0.001) and 2nd wave (β = –0.05, *p* < 0.001), which also holds for URLs (model 5) with stringency effects (β = –0.01, *p* < 0.001), interim (β = –0.08, *p* < 0.001) and 2nd wave (β = –0.05, *p* < 0.001).

**Table 2:** Linear mixed effects models for each meta path and for a composition of all types.

	**Hashtag**		**Mention**		**Named entity**	
**Coefficient**	**Estimate**	**Conf. Int (95%)**	**Estimate**	**Conf. Int (95%)**	**Estimate**	**Conf. Int (95%)**
Stringency Score (+10 p)	–0.001^***^	–0.002 to –0.000	–0.011^***^	–0.012 to –0.009	–0.002^***^	–0.002 to –0.001
Period (Ref. 1st wave)						
Period (Interim)	–0.003^**^	–0.006 to –0.000	–0.046^***^	–0.053 to –0.039	–0.010^***^	–0.012 to –0.008
Period (2nd wave)	–0.001	–0.003 to 0.000	–0.027^***^	–0.031 to –0.023	–0.001^*^	–0.002 to 0.000
Intercept	0.946^***^	0.906 to 0.987	0.957^***^	0.873 to 1.040	0.954^***^	0.896 to 1.012
**Random Effects**						
σ^2^	0.00		0.00		0.00	
τ_00_	0.01^*country*^		0.05^*country*^		0.02^*country*^	
ICC	0.98		0.98		1.00	
N	28^*country*^		28^*country*^		28^*country*^	
Observations	2,576		2,576		2,576	
Marg. R^2^ / Cond. R^2^	0.000 / 0.982		0.003 / 0.979		0.000 / 0.996	
BIC	-14076.202		–9947.884		–15897.409	
	**Retweet**		**Url**		**All Meta paths**	
**Coefficient**	**Estimate**	**Conf. Int (95%)**	**Estimate**	**Conf. Int (95%)**	**Estimate**	**Conf. Int (95%)**
Stringency Score (+10 p)	–0.012^***^	–0.014 to –0.009	–0.011^***^	–0.015 to –0.007	–0.007^***^	–0.010 to –0.005
Period (Ref. 1st wave)						
Period (Interim)	–0.067^***^	–0.075 to –0.059	–0.079^***^	–0.093 to –0.065	–0.041^***^	–0.050 to –0.032
Period (2nd wave)	–0.054^***^	–0.059 to –0.049	–0.047^***^	–0.055 to –0.039	–0.026^***^	–0.031 to –0.021
Meta path (Ref. Hashtag)						
Meta path (Mention)					–0.074^***^	–0.079 to –0.069
Meta path (Named Entity)					0.002	–0.003 to 0.008
Meta path (Retweet)					–0.113^***^	–0.118 to –0.107
Meta path (Url)					–0.267^***^	–0.273 to –0.262
Intercept	0.941^***^	0.861 to 1.021	0.784^***^	0.691 to 0.876	1.007^***^	0.941 to 1.073
**Random Effects**						
σ^2^	0.00		0.00		0.01	
τ_00_	0.04^*country*^		0.06^*country*^		0.03^*country*^	
ICC	0.97		0.92		0.75	
N	28^*country*^		28^*country*^		28^*country*^	
Observations	2576		2576		12,880	
Marg. R^2^ / Cond. R^2^	0.009 / 0.965		0.007 / 0.920		0.204 / 0.797	
BIC	–8948.098		–6157.597		–22621.614	

* *p < 0.1*,

** *p < 0.05, and*

*** *p < 0.01*.

Fitting a model for all meta paths (model 6), we found a statistically significant and negative effect for stringency (β = –7.21e-03, *p* < 0.001), negative effects for the interim (β = –0.04, *p* < 0.001) and the 2nd wave (β = –0.03, *p* < 0.001). Controlling for meta path type, we observe significant negative effects of mention (β = –0.07, *p* < 0.001), retweet (β = –0.11, *p* < 0.001) and URL (β = –0.27, *p* < 0.001) in comparison to hashtag-based discursive linkages. The full model's total explanatory power is substantial (conditional *R*^2^ = 0.80) and the part related to the fixed effects alone (marginal *R*^2^) is of 0.20. All in all, the mixed effects model clearly lends support to hypothesis 2, as stringent measures of crisis management taken at the domestic level seem to have structuring effects toward national discourse communities at the expense of the transnationality of COVID-19-related discourse on Twitter. The strength of the observed transnationality seems to also depend on the type of meta path with negative effects compared to retweet-based paths consistent with Figure 3.

### 6.3. Country Comparison

**Figure 4:**
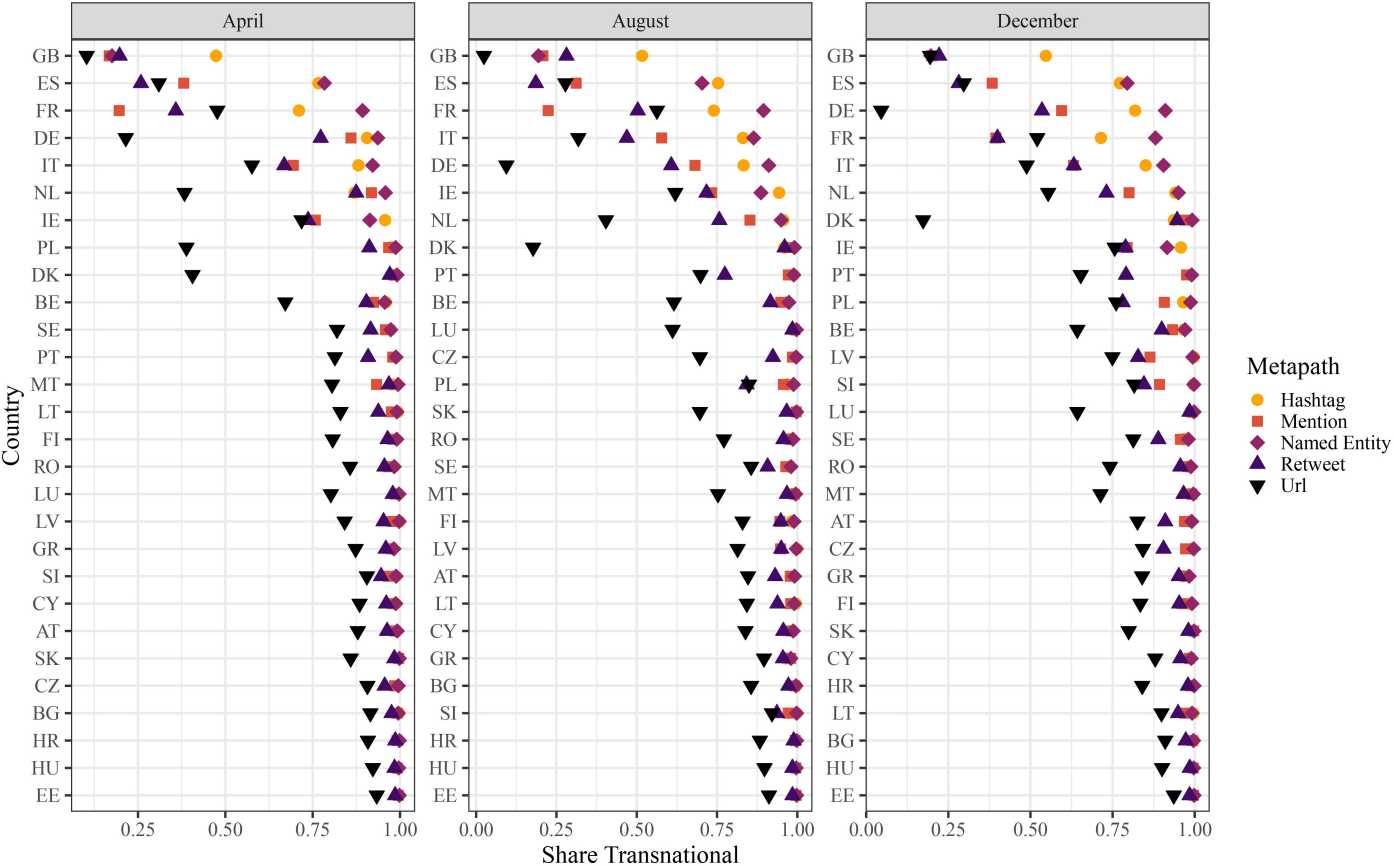
Country-wise shares faceted by month. The point shapes and colors shows the respective type of meta path. The countries are ordered according to their average share of transnational communication for each month individually.

Finally, we take a look at the country level by disaggregating our indicator per meta path and country (see Figure 4). We can observe a great variation in transnationality scores per country which seems to be related to the size of the population as well as the regional location. Over all meta path-specific indicators, smaller countries, especially the ones in Central and Eastern Europe, show higher shares of transnationality than larger countries. In contrast, variation is higher among the larger countries. Nevertheless, for all meta path-specific indicators, we find the lowest values for the United Kingdom, followed by Spain, France, Germany and Italy. This difference is not just produced by the amount of communication. Within our sample, we observe only a very small effect of the amount of communication (β = –4.069e-12, *p* < 0.001) if we include the number of paths in our final model (compared to Table 2). The reduction of the conditional R^2^ of just 0.002 indicates a substantial effect of country beyond its amount of twitter communication. With respect to usage patterns, countries whose Twitter populations make comparatively frequent use of @-mentions and retweets such as Spain, France and the United Kingdom (for a full overview see Supplemental Figure 3), which can be regarded as an indication of a higher tendency to use Twitter for formal news dissemination (Poblete et al., 2011), also tend toward more nationally structured COVID-19 related Twitter discourses. However, this relation does not seem consistent (see, for instance, Germany, Italy or the Netherlands). With respect to the subindicators, the national comparison reveals some additional insights. As already discussed with respect to the aggregate figures, we see that URLs produce the lowest shares of transnational discursive linkages for almost all countries. Notable exceptions here are France and—to a lesser extent—Spain, where indicators for retweets and @-mentions score lower than for URLs. Furthermore, @mentions and retweets score remarkably lower in general than hashtags and named entities. Finally, what has been assumed for named entities—that they more closely represent a national discourse and are thus responsible for less transnational patterns—seems to hold true only for the United Kingdom and less so for Spain, whereas transnationality scores for named entities are among the highest when looking at the other countries.

As to cultural globalization, we expected a positive correlation between the extent to which a country is globalized in socio-cultural terms with transnational linkage shares measured by our HIN-based methodology. Table 3 gives a Spearman's Rank Correlation of mean and median shares with the KOF Globalization Index. As the numbers show, effects are negative and mostly non-significant except for URLs. While this clearly puts H4 into question, it is important to note that the variables for Twitter usage (general purpose and news) from the Reuters Digital News Report show significant negative effects for almost all metapath-specific measurements. Table 3 shows the respective values. The findings suggest that Twitter usage indeed has an impact on our measurement, yet in the sense that the more Twitter is used by a population, the more nationalized its Twitter discourse appears to be. In contrast, smaller Twitter populations in a given country tend to be closer aligned to a transnational discourse throughout all the phases covered. This general observation is plausible given the probable greater bias toward elite actors for smaller Twitter populations, whereas larger Twitter communities represent larger parts of the population and thus more fully represent a nationally mediated public discourse.

**Table 3:** Country-wise rank correlation (Spearman's Rank Correlation) of mean and median shares with KOF Globalization Index 2019 (KOFGI) social dimensions, Reuters social media usage 2020 (any purpose/general usage); and Reuters social media usage 2020 (news) by country.

**Indicator**	**Metapath**	**Spearman mean**	**Spearman median**
KOFGI (Score)	Hashtag	–0.393	–0.394
	Mention	–0.336	–0.336
	Named entity	–0.354	–0.349
	Retweet	–0.263	–0.274
	Url	-0.354	–0.410^*^
Reuters general (%)	Hashtag	–0.562^***^	–0.572^***^
	Mention	–0.628^***^	–0.634^***^
	Named entity	–0.695^***^	–0.683^***^
	Retweet	–0.556^***^	–0.536^**^
	Url	–0.241	–0.242
Reuters news (%)	Hashtag	–0.634^***^	–0.650^***^
	Mention	–0.699^***^	–0.726^***^
	Named entity	–0.764^***^	–0.750^***^
	Retweet	–0.698^***^	–0.680^***^
	Url	–0.352	–0.348

* *p < 0.1*,

** *p < 0.05, and*

*** *p < 0.01*.

## 7. Discussion

As presented in the results section, our study indicates that transnational discursive linkages have increased over the course of the pandemic, at least in Europe over the first months after its appearance from spring to summer 2020. The general experience of a global community of fate, which COVID-19 might have evoked, is thus partly supported by slight trends of discursive alignment across national borders via digital media. However, this overall statement needs to be differentiated in a number of relevant ways.

First, our research could not establish a general relationship between the transnationality of Twitter discourse and the severity of the pandemic, which had been our initial hypothesis (H1). What we have observed instead is an upward movement, starting at a comparatively low level for the first wave and then growing toward the interim period and stagnating (or even slightly declining) in the second wave. There is only one entity-specific subindicator—URLs—, for which we found the expected pattern of a U-curve with more transnational discursive linkages during the subsequent waves of the pandemic compared to less transnationally shared content during the interim period. This is telling, as among our multidimensional entity classes, URLs are the ones that arguably open a window to wider discursive universes and thus, in contrast to platform-specific entities, allow to go beyond the Twitter environment while not being as generic an indicator of discursive linkage as named entities. In previous research, sharing URLs has been perceived as an indication for more formal news dissemination in Twitter communication (Poblete et al., 2011). Thus, we can generally assume URLs to be bound more closely to a nationally structured public sphere and discourse, reflecting the prominent role of legacy media organizations. From this perspective, it makes sense that the transnational sharing of web sources is more frequent when the pandemic as a global event is salient than in periods of relative calm, when the global disease is only one topic among others. Another explanation for the unexpectedly high shares of transnational linkages during the interim period might be the fact that seasonal effects of the pandemic affect world regions differently so that, for instance, Twitter users in European countries might discuss dynamic developments on the Southern hemisphere, while going through a time of relative calm themselves.

Through our temporally sensitive design we have been able to observe correlations with political activities of crisis management. All stringent measures of crisis management were adopted and implemented at domestic level, reflecting an institutional nationalism apparently still dominant when it comes to public health or civil protection. With respect to the transnationality of COVID-19 related discourse, stringent measures had the expected effect of reinforcing national structuration of discourse and thus cause decreases in transnational linkage shares. Therefore, our multi-level analysis lends clear support to hypothesis 2.

Another important point to reflect upon when interpreting the results would be that April 2020, while serving as the natural starting period of our chronological observation, likely constitutes the most exceptional period covered in our research. With the first news on European incidents and casualties, and unprecedented political measures like general lockdowns imposed on populations across Europe and the world, people found themselves in a state of exception. It has likely taken time for routines of social exchange and communication to be reactivated—with the particular help of digital communication media. Thus, what we might observe in our analyses is how people learned to better cope with the novel situation caused by the pandemic and crisis management measures, including how to uphold connectivity and discourse across borders. Such ideas of adaptation and learning coincide with our third hypothesis. All in all, our empirical results lend support to H3: The share of transnational discursive linkages has increased over the course of the pandemic. Our combined indicators suggest that Twitter user populations across Europe have found more coherent ways to discursively deal with the pandemic and the socio-political effects it produced.

As to international variation and hypothesis 4, we do not find support for our basic expectation that transnational discursive linkages depend on the extent to which a national society is globalized. On the contrary, for URLs, we see a significant negative correlation (Spearman's Rank) between the KOFGI and our transnationalization measure. This might be explained with the fact that larger countries are likely to be more self-sufficient in professional news dissemination than smaller countries. This might be of particular importance during a pandemic with the general dependence on high quality information on public health. The other, non-significant KOFGI correlations indicate a similar direction. Moreover, Twitter usage seems to be an intervening factor, as transnationality of COVID-19 related Twitter discourse negatively correlates with Twitter usage both for any purpose and for news. This makes sense with respect to the differently skewed representation of national samples (Mellon and Prosser, 2017). Therefore, one should expect the general public discourse to be better represented in the COVID-19 related discourse of larger Twitter populations such as—most particularly—the United Kingdom, but also France or Spain. In contrast, smaller samples mostly include elite-level communication, showing a closer alignment to global, transnational communicative patterns.

A number of limitations of our research and constraints in interpretability need to be considered. First of all, while the levels of transnational shares that we observe for our entity-specific indicators differ considerably, making judgements about whether they have generally reached a high or low level is not trivial. While for the most transnational entity type, hashtags, they range between impressive shares of 60 to 80 percent, they are remarkably lower for other entities. Especially for URLs, the share of transnational linkages is strikingly low. On some days during the interim period, transnational shares for URLs are close to zero. Moreover, when disaggregating by country, we see that ranges for some countries are even wider and minima are lower for large countries, especially for Great Britain. Thus, from a macro perspective, while we measure transnational discursive linkages and their tendency to increase, there are still strong indicators for national discourse structuration in the COVID-19 related Twitter discourse that can be explained by important macro-level factors such as language, culture and proximity.

All our findings need to be taken with a grain of salt given that our dataset—as many others in the field of Twitter research—suffers from a number of biases related to the self-selection effects of Twitter usage as presented in subsection 2.4. Of highest relevance to our comparative study is the lack of representation with respect to societies and languages. Even though we deliberately selected a multilingual dataset, the vast majority of tweets covered are in English. Data collection via hashtags and other pre-selected terms have likely favored English-speaking countries as well. Finally, also during data processing and entity extraction, it is very likely that algorithms such as geolocation or named entity recognition work better for English, and thus produce more and better results here than for all the other idioms.

A further limitation of our research design that affects possible explanations is the lack of a plausible baseline for our temporal comparison. This is a frequent issue for both research on transnationalization and Twitter research as such, given that appropriate longitudinal datasets are often not available. This is evidently true for our research as well, as there simply was no substantial COVID-19 related Twitter discourse before the beginning of 2020. Given the great variety of *ad-hoc* issue publics on Twitter, it would also not have made sense to compare indicators against some sort of random sample for which similar processing (including geolocation, entity extraction and annotation) would have not only been prohibitively time consuming, but unclear in its comparability. Instead, we chose a different path, conceiving the interim period between both waves of the pandemic as a relative reference period. While we find this decision still very plausible, it of course affects our interpretation with respect to hypothesis 3 on adaptation and learning. Does the upward movement of transnational linkage shares from the first wave to the interim indicate a COVID-19 effect on transnational discourse that can be understood without considering the exceptionality of this first wave? Or is this development just reflecting a form of normalization after the initial stage of paralysis? This question must remain open for future research as we do not have the proper benchmark or longitudinal data on which we could base the interpretation of our findings.

What is true for the comparison with respect to the past can also hold for the future, as we do not clearly see where all this leaves us in the long run. What can be taken for granted is that the pandemic and travel restrictions generally served as a driver for the expansion of digital communication, including cross-border communication. Whereas, in previous ages diseases reduced social communication due to their dependency on physical mobility, this fundamental connection seems somewhat resolved by digitalization. The pandemic and our adaptation of new rules for social life have given clear proof that digital platforms can provide substitutes for most forms of social communication and discourse. However, this should not make us neglect the potential effects that the drastic reduction of physical mobility, especially across borders, might cause in the long run. Given the fact that international travel has served as important driver for transnational connectivity over decades, it is likely that a substantial reduction can have the opposite longitudinal effect.

## 8. Conclusion

In this paper, we presented a novel HIN-based methodology for studying transnational discursive linkages in issue publics on Twitter. The COVID-19 pandemic served as a background context that motivated our issue-oriented interest. Thereby we contribute to the current research on the social effects of this extraordinary global crisis. We applied our method to a subset of TBCOV, a uniquely rich multilingual dataset of geolocated tweets. Focussing our regional scope on Europe helped us to avoid unrealistic expectations and relates our research to the ongoing quest for a European public sphere and the empirical research devoted to this question. Our findings suggest that the coherence of COVID-19 related Twitter discourse has not been a function of the severity of the pandemic, which would have supported the metaphoric understanding of the pandemic as building a community of fate, but that it interacts in more complicated ways with structuring factors that tend to conserve the pre-existent communities of place. What we observe is that transnational discursive coherence grows over the first months of the pandemic. However, this upward movement was cut, with our indicator remaining at a stable level between summer 2020 and the second wave in December. While adaptation to the pandemic context seems to increase transnational discursive linkages, a steady growth is arguably hampered by structural conditions. One factor that we studied more closely were the stringent measures of crisis management taken at domestic level. These had nationalizing effects, reducing the shares of transnational linkages significantly around such regulatory events. Moreover, we have found interesting variations with respect to the linkage types included in our measurement as well as for the heterogeneous set of European countries. These insights into the complex geography of Twitter are also valuable for future researchers. While we discussed the limitations of our research in depth in Section 7, conclusions to be drawn from our study are particularly limited by its regional scope. Future studies should widen the scope beyond Europe or other regions, as only a global perspective would allow to reveal the structuring effects that regions itself (like Europe) have on the patterns of transnational exchange and discourse.

## Data Availability Statement

The datasets used in this paper are available via the original providers. TBCOV: https://github.com/CrisisComputing/TBCOV; COVID-19 policy response tracker: https://github.com/OxCGRT/covid-policy-tracker; KOF Globalization Index: downloadable file, URL: https://kof.ethz.ch/prognosen-indikatoren/indikatoren/kof-globalisierungsindex.html; Reuters Digital News Report: full datasets upon request, for further information see: https://reutersinstitute.politics.ox.ac.uk/digital-news-report/2021/resources. The original contributions presented in the study are included in the article/Supplementary Material, further inquiries can be directed to the corresponding author.

## Author Contributions

WS, AB, JZ, and TK contributed to conception and design of the study. WS was mainly responsible for theoretical foundations, including conceptions and metapath specifications, and wrote the first draft of the manuscript. AB and TK organized the database. AB and JZ performed the network analysis and wrote sections of the manuscript. All authors contributed to manuscript revision, read, and approved the submitted version.

## Funding

This research has been funded by the Klaus Tschira Foundation in the framework of the EPINetz project. We acknowledge financial support by Stiftung Universität Hildesheim.

## Conflict of Interest

The authors declare that the research was conducted in the absence of any commercial or financial relationships that could be construed as a potential conflict of interest.

## Publisher's Note

All claims expressed in this article are solely those of the authors and do not necessarily represent those of their affiliated organizations, or those of the publisher, the editors and the reviewers. Any product that may be evaluated in this article, or claim that may be made by its manufacturer, is not guaranteed or endorsed by the publisher.

## Footnotes

^1^While bans have multiple reasons, we found the most occurring non-dehydratable tweets based on bans of persons of interest like Donald Trump, Katie Hopkins or (Covid-related) disinformation accounts. With the ban of such accounts, tweets referencing them via e.g., mentions could not be rehydrated for our analysis. Additionally, retweets in our sample are restricted to API-delivered tweet contents due to computational restrictions.

^2^https://github.com/OxCGRT/covid-policy-tracker/blob/master/documentation/codebook.md; last accessed: 22-02-22.

## Supplementary Material

Supplementary Figure 1 Stringency of measures and transnational share of meta paths on a monthly comparison. Colors describe mean (red points and lines) and median (blue points and lines). Linetype and shape are used to differ between stringency index (dotted) and transnational share (solid).

Supplementary Figure 2 Country-wise development of stringency index. The lines shows the index development per country, facetted by month.

Supplementary Figure 3 Mean number of entities per tweet by country for all indicator (top subfigure) and facetted by entity type (bottom subfigure).

Supplementary Table 1 Sum of meta paths per Type and Month.
