# Supplementary figures and images for: Leveraging Dynamic Heterogeneous Networks to Study Transnational Issue Publics. The Case of the European COVID-19 Discourse on Twitter
Source: Front Sociol. 2022 Jun 30;7:884640. doi: 10.3389/fsoc.2022.884640 (PMC9280175; doi:10.3389/fsoc.2022.884640)

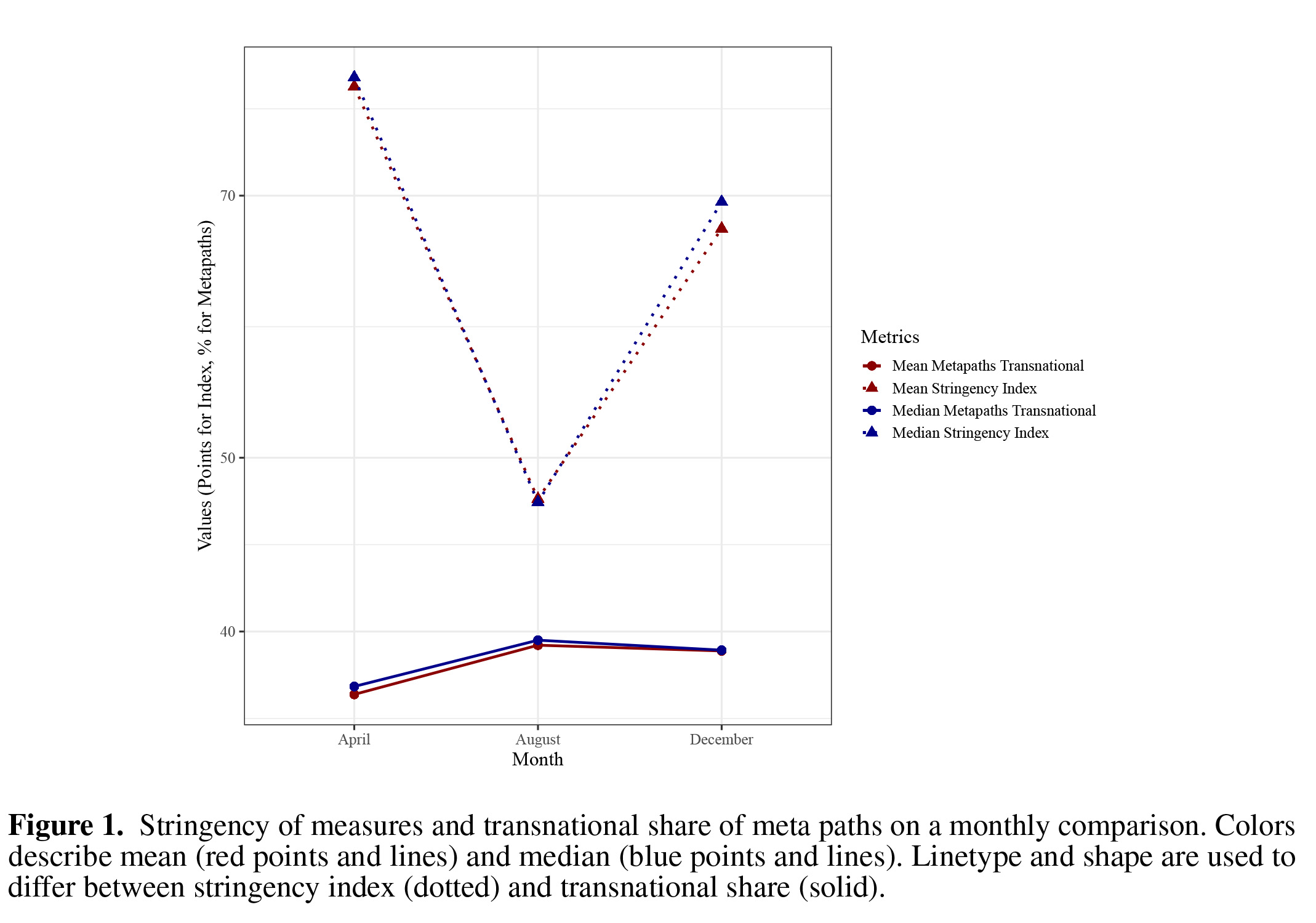

Supplement: Supplementary Figure 1 — Stringency of measures and transnational share of meta paths on a monthly comparison. Colors describe mean (red points and lines) and median (blue points and lines). Linetype and shape are used to differ between stringency index (dotted) and transnational share (solid). [file Image_1.jpg]

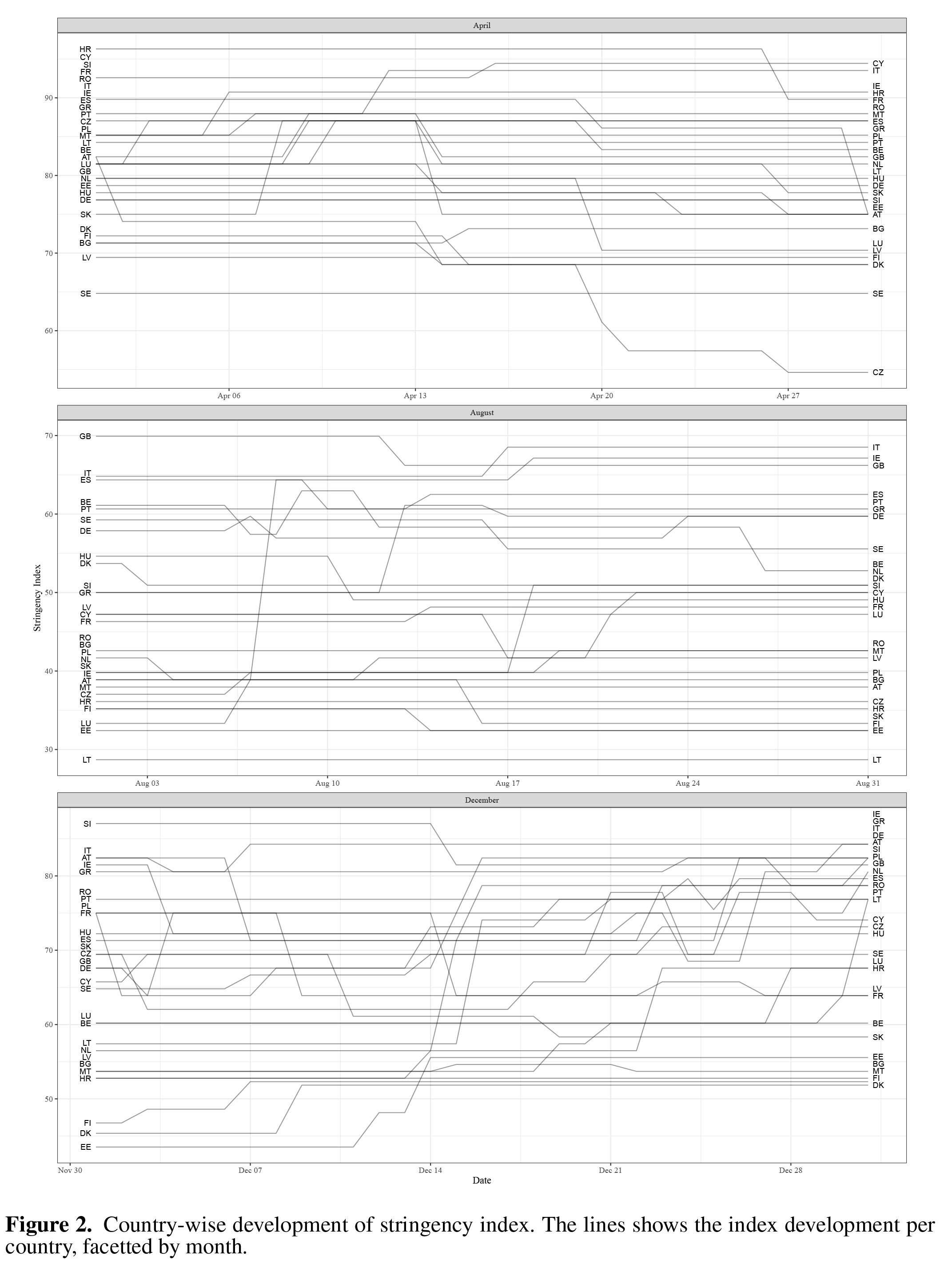

Supplement: Supplementary Figure 2 — Country-wise development of stringency index. The lines shows the index development per country, facetted by month. [file Image_2.jpg]

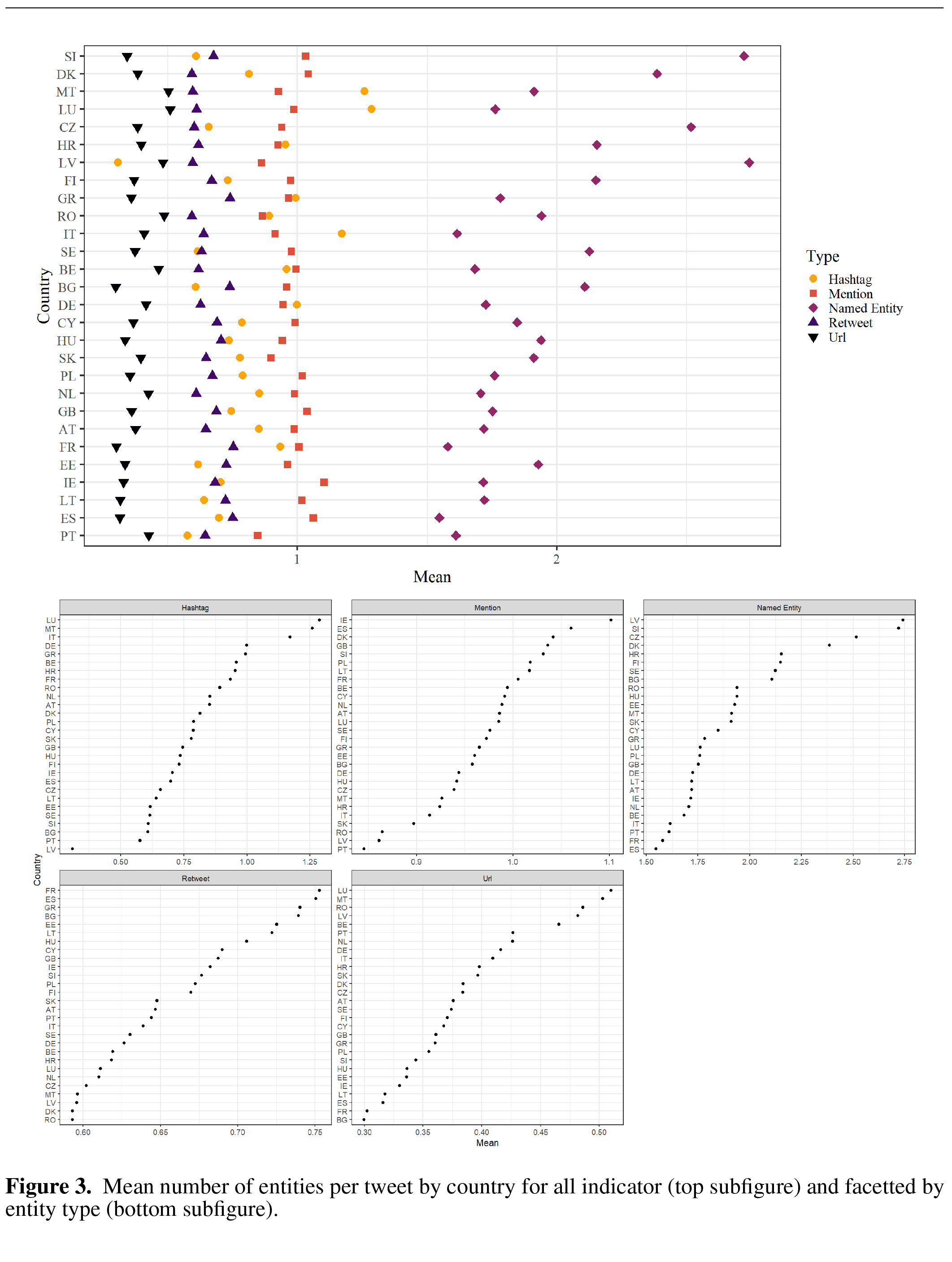

Supplement: Supplementary Figure 3 — Mean number of entities per tweet by country for all indicator (top subfigure) and facetted by entity type (bottom subfigure). [file Image_3.jpg]
