# Supplementary material for: Leveraging Dynamic Heterogeneous Networks to Study Transnational Issue Publics. The Case of the European COVID-19 Discourse on Twitter
Source: Front Sociol. 2022 Jun 30;7:884640. doi: 10.3389/fsoc.2022.884640 (PMC9280175; doi:10.3389/fsoc.2022.884640)
Supplement: Supplementary Table 1 — Sum of meta paths per Type and Month. [file Data_Sheet_1.pdf]

1 APPENDIX

Table 1. Sum of meta paths per Type and Month

Month	*meta path type*	$\sum$
April	Hashtag	183,152,773,817
August	Hashtag	71,807,356,838
December	Hashtag	102,002,434,442
April	Mention	88,210,966,791
August	Mention	42,672,985,372
December	Mention	58,087,700,278
April	Named Entity	1,939,063,021,718
August	Named Entity	937,127,021,026
December	Named Entity	1,788,033,469,916
April	Retweet	2,975,108,151
August	Retweet	1,025,944,555
December	Retweet	918,763,562
April	Url	474,629,025
August	Url	376,811,772
December	Url	183,073,126
